# Elaboration of Cookies Using Oils and Flours from Seeds and Nuts: Effects on Technological, Nutritional and Consumer Aspects

**DOI:** 10.3390/foods11152249

**Published:** 2022-07-28

**Authors:** Elena Martínez, Manuel Álvarez-Ortí, Adrián Rabadán, Cristina Millán, José E. Pardo

**Affiliations:** Higher Technical School of Agricultural and Forestry Engineering, University of Castilla-La Mancha, Campus Universitario, s/n, 02071 Albacete, Spain; elena.martinez@uclm.es (E.M.); manuel.alvarez@uclm.es (M.Á.-O.); adrian.rabadan@uclm.es (A.R.); cristina.millan@uclm.es (C.M.)

**Keywords:** cookies, food reformulation, seeds, nuts, functional food, food neophobia

## Abstract

The consumption of cookies is widely extended throughout the world, although their formulas contain ingredients such as saturated fats or refined flours that are considered harmful to health. In addition, cookies are generally made from wheat flour, and nowadays there is a growing concern about gluten intolerance, thus the demand for gluten-free products is increasing. In this regard, the aim of the present study is to reformulate traditional cookies by replacing wheat flour and butter by flours and oils from nuts and seeds. Within these seeds, poppy or chia are not commonly consumed ingredients as they can cause rejection by consumers. Thus, a study was performed to evaluate the neophobia level of consumers and the consumer acceptance for the inclusion of these novel ingredients in cookies. The results have been obtained by measuring physical parameters, proximate composition and consumer evaluation of five batches of cookies. By replacing butter and wheat flour with maize flour, almond, walnut, chia or poppy seed flours and oils, an increase of protein, fat and fiber has been observed as well as a decrease in carbohydrate content; thus, the resultant cookies would be a good source of vegetal protein as well as a source of oleic and linoleic acid with potential benefits on health. The cookies in general have similar physical properties and a positive consumer acceptance in texture, taste and external aspect. The Food Neophobia Scale results suggest that non-neophobic consumers gave higher scores than neophobic consumers in all the parameters. The resultant product would be a functional product able to substitute traditional ones not only directed to celiac people but all type of consumers because of their beneficial composition.

## 1. Introduction

Cookies are a very popular baked snack characterized by a formula in which sugar, shortening and wheat flour are the main ingredients [1]. The shortening consists of fat which must remain in a solid state at room temperature, thus saturated fatty acids predominate its composition. There are many adverse health effects associated with the excessive consumption of saturated fatty acids, mainly derived from its incidence in cardiovascular diseases [2]. The concern of today’s society for a healthy and balanced diet makes the food industry investigate new functional products to meet these new demands. Functional food is a natural or processed food that presents active compounds in defined quantitative and qualitative amounts that provides a documented health benefit [3].

One of the main objectives of the agri-food industry is to elaborate healthier foods. In the case of cookies, which are one of the most consumed snacks worldwide, especially for children, the elaboration of a healthier alternative to traditional cookies consists of the partial elimination of harmful ingredients such as butter or wheat flour and the introduction of other beneficial ingredients that contain nutrients such as fiber, protein or unsaturated fatty acids that can improve the health of the general population and help to achieve a better cognitive development in children.

Within this frame, it is desirable to replace these saturated fats with other oils that have a higher percentage of unsaturated fatty acids in their composition. In this sense, vegetable oils extracted from nuts and other seeds are generally rich in unsaturated fatty acids. This is the case of walnut oil, which is especially rich in linoleic acid [4], or chia oil, characterized by its high content in linolenic acid [5]. In addition, nut and seed oils contain many other bioactive compounds with antioxidant capacities, such as tocopherols or phenolic compounds [6].

On the other hand, cookies are generally made from wheat flour due to the viscoelastic properties of gluten. This protein is responsible for celiac disease, which can be defined as an autoimmune inflammatory disease that causes gastrointestinal disorders resulting in a deficient nutrient absorption [7]. Celiac disease has become one of the most frequent food intolerances in many parts of the world. Thus, the use of gluten-free flours as an alternative to wheat is a challenge for the food industry, which may elaborate products adapted to the needs of people with celiac disease. In this sense, partially defatted flours from nuts and seeds that are a byproduct of the oil extraction industry have proven to be a novel useful ingredient to be included in cookie formulation [8] and may also be a vehicle to enhance the nutritional properties of cookies [9]. However, it is important to evaluate the behavior of the dough made with gluten-free flours during the baking process to ensure that the textural properties of the cookies remain the same as the ones elaborated with wheat [10].

Partially defatted almond flour is rich in proteins, which could positively influence the structure of the dough when baking, thus it would be an interesting ingredient to replace conventional flours [11]. This flour also contains a high concentration of vital mineral such as zinc and iron and, compared with wheat flour, it shows a higher content of Ca, Fe, Na, K, Zn, Cu and Mg [12]. Walnut flour is a good source of amino acids such as aspartate, glutamate and arginine, and minerals such as calcium and phosphorous are present in higher amounts compared with wheat and rice flours [13]. On the other hand, partially defatted seed flours are other gluten-free flours that can be used to replace wheat flour with advantages in terms of their nutritional components. In this sense, chia flour contains many antioxidants, minerals and vitamins that contribute to correct intestinal functioning and decrease blood cholesterol and glucose levels [14]. Poppy flour is rich in protein, carbohydrate, dietary fiber and bioactive compounds [15].

The main problem with the introduction of these seeds and nuts is that they could affect the external appearance and taste of the cookies compared with cookies elaborated with wheat flour. Since chia and poppy flours are black, it is necessary to add small quantities to avoid excessive changes in the external appearance. In addition, due to their high fiber content, the percentage used should be small in order to not have to limit its consumption. Moreover, in the case of nut flours, walnut flour is characterized by a great astringency, thus its addition must be controlled to prevent the alteration of cookies’ flavors. On the contrary, almond flour color is like wheat flour and the taste and smell is mild and pleasant. However, none of these flours can be added without the addition of maize flour, because it is necessary to be able to manipulate the dough to obtain a softer cookie. Rai et al. [16], used a combination of different flours to evaluate the behavior changes by adding different combinations of flours following the same cookie recipe. They reported that the texture, the spread factor and the nutritional value changed, thus different combinations of gluten-free flours must be analyzed to produce good quality cookies with acceptable physical and sensory qualities.

Considering the increased incidence and prevalence of diseases closely linked to food, the objectives of the study were to evaluate the effect of the inclusion of nut and seed oils as well as gluten-free flours in the formulation of cookies. These effects must be analyzed for their physical, nutritional and sensory parameters in order to obtain healthier products with an improvement in their nutritive characteristics and to be well accepted by the consumers. Due to the color and/or the flavor of the flours and oils from nuts and seeds, a combination of different nuts and seeds has been used in order to obtain cookies similar to the traditional recipe and to evaluate the possible nutritional improvement, especially in the content of fiber, protein or fat. Thus, the main objective of this work is to improve the nutritional characteristics of a very popular snack, especially for children, by trying to use novel ingredients to change potentially harmful compounds such as saturated fatty acids for unsaturated fatty acids reported to be beneficial for health.

## 2. Materials and Methods

### 2.1. Raw Materials

The wheat and maize flour, the butter, the seeds of chia (*Salvia officinalis*) and poppy (*Papaver somniferum*), the eggs, the sugar, the salt, the milk, the almonds and the walnuts were purchased in local markets.

The oils used were obtained by mechanical processes. The walnuts and almonds were first ground and then subjected to a pressure of 200 bar for 10 min in a hydraulic press (Mecamaq DEVF 80, Vila-Sana, Lleida, Spain) [17]. Chia and poppy seeds were subjected to oil extraction with a screw press Komet Oil Press CA59G (IBG Monforts Oekotec GmbH & Co. KG, Mönchengladbach, Germany), where the barrel was previously heated to 100 °C.

The different defatted flours from almond, walnut, chia and poppy used in the elaboration of cookies were obtained by grinding the press cake resulting from the oil extraction process and sifting through a sieve of 1 mm.

Table 1 shows the nutritional composition of the flours used for the formulation of the cookies. Table 2 refers to the chemical composition of the oils of the nuts and seeds used.

As wheat and maize flours were acquired in a local market, the nutritional value was obtained from the label of both flours. Wheat flour is composed of 1.2% fat, 75% carbohydrates and 9% protein. Maize flour is composed of 0.5% fat, 86% carbohydrates, 0.5% protein and 1% fiber.

### 2.2. Cookies Formulation

The cookies were elaborated from a dough formed with the following ingredients: 250 g flour, 125 g sugar, 80 g egg, 150 g fat, 15 g milk, 1 g salt. The flour and fat source were changed as shown in Table 3. To elaborate the cookies, first the fat and sugar were mixed. Then, the milk and the egg were added and mixed again. Finally, the flour and the salt were added and kneaded until a malleable and homogeneous dough was formed. The dough was rolled to a thickness of 5 mm and cut into 5 cm diameter circular pieces. Finally, the cookies were baked at 180 °C for 15 min and stored under vacuum conditions and refrigerated until evaluation.

The inclusion of oils from nuts and seeds instead of butter can affect the flavor and the palatability of the cookies and can be perceptible by the consumers. In the case of chia, as it is rich in linolenic acid (omega-3), when cooked it can be perceived with a slight fishy taste, thus it was not used in the formula for the cookies. In addition, poppy seed oil presents a strong flavor that masks the flavor of the cookie if added in large quantities, thus that is the main reason to mix it with other nut oils. In the case of nut oils, both present pleasant flavors, but it is interesting to mix the walnut with the almond, because the walnut oil flavor is stronger. Finally, butter and walnut oil have been combined at 50% to make the cookie more similar to the traditional one.

### 2.3. Physical Parameters

The diameter and the thickness were measured in 5 baked cookies from each batch with a digital caliper. For the diameter, the mean was calculated from two perpendicular measures of each cookie. The expansion factor was calculated by dividing the diameter by the thickness.

The color was measured by reflection in three points on the surface of the dough and the baked cookies with a colorimeter (Minolta CR-200, Minolta Camera Co. Ltd. Osaka, Japan), using the illuminant D-65. The CIELab chromatic coordinates a* (red–green component) and b* (yellow–blue component) were annotated as recommended by the International Commission on Illumination [18]. The measurements were repeated 3 times for each different cookie sample.

To measure the texture of the cookies, five cookies of each type were cut perpendicularly with a Warner Bratzler blade with a rectangular slot blade at the constant velocity of 2 mm/s, using a TA-XT Plus texture analyzer (Stable Micro Systems, Godalming, UK). The maximum force that was needed to cut the cookie and the deformation before breaking were recorded.

### 2.4. Proximate Composition

The method used to determine the water content consisted of measuring the weight loss after drying at 103 °C until reaching constant weight [19]. The ash content was determinate by calcinating the samples at 550 °C until reaching constant weight [19].

Crude fat was estimated gravimetrically by the filter bag technique after the petroleum ether extraction of the dried sample in an Ankom XT10 extraction system [20]. Protein content was measured by multiplying by a conversion factor of 6.25 the total nitrogen content [21]. Crude fiber was measured by using the Weende technique adapted to the filter bag technique [20].

The total carbohydrate content was calculated by subtracting the sum of the contents of the crude protein, fat, water and ash of the total weight of the sample [22], and the available carbohydrate content was calculated by subtracting the crude fiber of the total carbohydrate content [23]. The energy value was estimated from the relative contents of the protein, fat and carbohydrates, applying Atwater factors of 4.0 kcal/g for protein, 9.0 kcal/g for fat and 4.0 kcal/g for carbohydrates.

### 2.5. Consumer Evaluation

To evaluate the acceptance of the cookies, an affective test was used. It was carried out in the sensory analysis laboratory at the Higher Technical School of Agricultural and Forestry Engineering in Albacete (Spain). A total of 103 consumers were asked to evaluate the external aspect, the texture and the taste of baked cookies using a nine-point scale ranging from −4, least preference (completely dislike), to +4, most preference (completely like) [24]. The sensory analysis was carried out with untrained consumer judges, who met the only condition of being regular cookie consumers.

In addition, consumers fulfilled a questionnaire related to their degree of food neophobia. A 10-item Food Neophobia Scale was used [25]. Consumers were asked to indicate their degree of agreement on a 7-point Likert-type scale, being ‘disagree completely’ (1) and ‘agree completely’ (7) about the statements stated on Table 4. The score is the sum of the 10 items of the scale after reversing the negative items (theoretical range 10–70). The highest scores indicate more food neophobia as well as a lower tendency to try new foods. Cronbach’s Alpha was calculated to evaluate the internal consistency of the scale.

### 2.6. Statistical Analysis

Results are expressed as means and all the parameters were measured at least in triplicate. To determine the statistical differences, the analysis of variance test (ANOVA) at a 5% level of significance was used, as well as the Duncan test (*p* < 0.05). In the food neophobia study, the T-test was applied (*p* < 0.05) to compare the sensory results between the considered neophobic consumers and the non-neophobic consumers. All statistical analyses were carried out using SPSS software (IBM SPSS Statistics, Windows v. 26, IBM Corp., Armonk, 241 NY, USA).

## 3. Results and Discussion

### 3.1. Physical Analysis

The cookie spread ratio is an important factor for evaluating the rising ability of cookies, since cookies with a low-spread ratio tend to rise better than those with a high-spread ratio. A higher spread factor and a larger diameter are important factors linked to the quality of the cookies. The spread factor of cookies is related to the quality of the flour used to elaborate cookies and depends mainly on the values of their thickness and diameter [26]. The final dimensions of the cookie and the related spread factor are important parameters in estimating the behavior of the dough during baking and depends largely on certain nutritional compounds of the cookie, such as the gluten, the sugar or the fiber content [27]. Table 5 shows the diameter, thickness and spread factor of the cookies.

The spread factor varied from 7.70 in the control sample to 11.91 in the walnut and chia cookies (AWC). The lowest spread factor was observed in the control sample, which means that this cookie, elaborated with wheat flour, grew less in diameter but more in thickness and may be because of the gluten that forms a web during the baking of cookies that increases viscosity and decreases the flow of mass, which causes a smaller diameter [28]. The diameter was larger in the reformulated cookies because of the lack of gluten in the formula, although the flours are rich in protein. Regarding the thickness, AWC and AWB presented the lowest values compared with the control, which correlates with the values obtained in the spread factor.

Color is one of the main attributes in terms of the physical characteristics of food and, in general, one of the first that consumers notice and value. For this reason, it is important to determine the possible changes in color due to the inclusion of new ingredients in order to verify that the final product has similar characteristics. The addition of new ingredients in the formulation can affect the values for a* and b* components.

**Figure 1:**
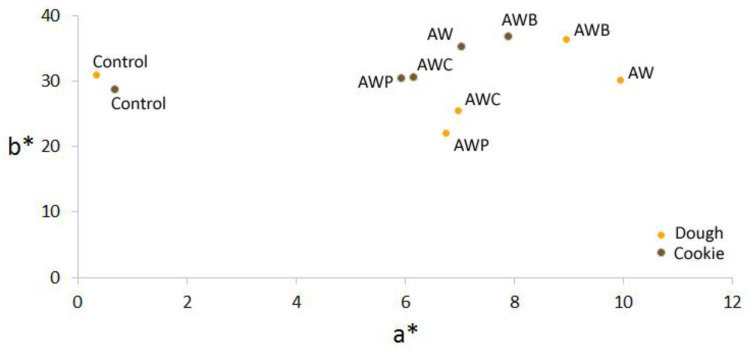
Color parameters of the dough and cookies elaborated with the different formulations. Values of the components a* (red–green) and b* (yellow–blue). Control—wheat flour (100%) and butter (100%); AWP—maize flour (50%), almond flour (33%), walnut flour (14%), poppy flour (3%), walnut oil (70%) and poppy oil (30%); AW—maize flour (60%), almond flour (28%), walnut flour (12%) and walnut oil (100%); AWC—maize flour (60%), almond flour (23%), walnut flour (12%), chia flour (5%), almond oil (50%) and walnut oil (50%); AWB—maize flour (82%), walnut flour (12%), almond flour (6%), butter (50%) and walnut oil (50%).

The control sample presented the lowest values of components a* and b* and, in general, the dough presented higher values compared with the baked cookies (Figure 1). However, the use of defatted flours from nuts or seeds causes very striking changes in the color of the cookies, which can be resumed in an increase in the values of the a* and b* component that was also described by [29] due to a difference in the natural pigments of nuts and seeds compared with wheat flour. Differences can be perceived between the dough and the cookie. Higher values for the component a* and b* were shown in the baked cookies and may be due to a change in the pigments of the oils and the flours when cooked.

**Figure 2:**
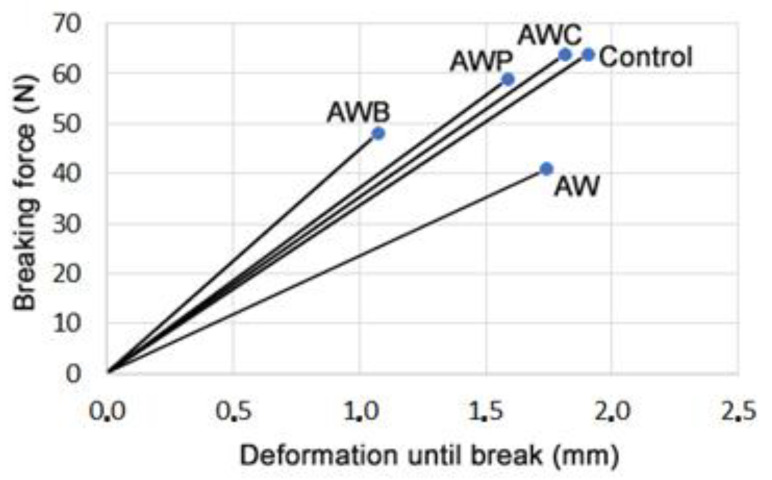
Breaking force and deformation until break of the cookies elaborated with the different formulations. Control—wheat flour (100%) and butter (100%); AWP—maize flour (50%), almond flour (33%), walnut flour (14%), poppy flour (3%), walnut oil (70%) and poppy oil (30%); AW—maize flour (60%), almond flour (28%), walnut flour (12%) and walnut oil (100%); AWC—maize flour (60%), almond flour (23%), walnut flour (12%), chia flour (5%), almond oil (50%) and walnut oil (50%); AWB—maize flour (82%), walnut flour (12%), almond flour (6%), butter (50%) and walnut oil (50%).

As regards texture, it is another important characteristic that the consumer values in the cookies. They must be crispy to be well valued by consumers. The analytical evaluation of cookies texture is difficult, since the results obtained are highly variable and difficult to interpret. However, the evaluation of the changes in the textural behavior can be analyzed by measuring the following two parameters: the maximum force, which measures the force needed to break the cookie, and the deformation that occurs in the cookie when the force is applied until it breaks (Figure 2). Regarding these two parameters, the cookies showed different behaviors. The control sample showed a harder texture, although the force needed to break the cookie was similar to samples AWC and AWP. On the other hand, sample AWB showed a softer behavior, since the breaking force was smaller, and sample AW showed a more brittle texture, as the slope of the curve shown on Figure 2 is lower, indicating that less force is needed to produce the cookie deformation. In general, previous works have shown that cookies formulated with partially defatted seed flours showed a more fragile behavior [30].

### 3.2. Proximate Composition

The results of the proximate composition of the different cookies elaborated are shown in Table 6. The moisture content measures free and bound water in the product. The moisture content is higher when nut and seed oils and flours are added and is also higher than the average content stated by other authors [31,32], which is around 5%. The main problem is that moisture is one of the parameters that affects the shelf life of cookies [33] and it can also be related to a less crispy texture.

The use of almond and walnut flour in the formulation of cookies resulted in a significant increase in the protein content related to the control cookies elaborated with wheat flour or cookie AWB elaborated with 70% maize flour. This was also reported by [34], who showed that, depending on the quantity and the type of flour used, the protein content increased or decreased according to the nutritional value of the seeds and nuts used.

The inclusion of chia defatted flour in the formulation of cookies (AWC), although in a small percentage, significantly increased the crude fiber content of the cookies. Chia seeds have 22.00 g/100 g of dietary fiber [35], which provides many health benefits such as improving serum lipid concentration or reducing the risk of developing different diseases such as coronary heart disease, stroke or hypertension [36].

Crude fat was higher in the cookies where no butter was added (AWP, AW, AWC). Yildiz and Gocmen (2020) [37] showed that reformulated cookies with almond presented higher values than the control sample maybe because of the presence of water in the butter that is not present in oils. In addition, the use of defatted flours from nuts or seeds may be another factor that increases the fat content, since, although these are byproducts of the oil extraction industry, they still contain a considerable amount of fat. The percentages of remaining fat in the flours are: chia flour around 11.67%, poppy flour around 11.52%, almond flour around 21.11% and walnut flour around 13.43%, which increase the fat content obtained in the proximate composition of the cookies. However, the nutritional quality provided by the oil and the flour of seeds and nuts is better than that of butter in relation to the content of unsaturated fatty acids, which have been reported to be beneficial to human health.

In the case of carbohydrates, the control sample and AWB that contain high amounts of maize flour in their composition present a higher carbohydrate value than cookies reformulated with nuts and seeds. Regarding these data, cookies reformulated with nuts and seeds would be an alternative source of protein and fiber with reduced carbohydrate content and containing fats that are beneficial for human health without hardly raising the energy value.

### 3.3. Consumer Evaluation

**Figure 3:**
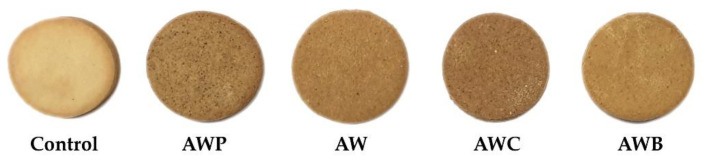
External aspect of the evaluated cookies. Control—wheat flour (100%) and butter (100%); AWP—maize flour (50%), almond flour (33%), walnut flour (14%), poppy flour (3%), walnut oil (70%) and poppy oil (30%); AW—maize flour (60%), almond flour (28%), walnut flour (12%) and walnut oil (100%); AWC—almond flour 823%), walnut flour (12%), chia flour (5%), almond oil (50%) and walnut oil (50%); AWB—maize flour (82%), walnut flour (12%), almond flour (6%), butter (50%) and walnut oil (50%).

Sensory tests were made to evaluate consumer preference regarding the external appearance of the cookies, the texture and the taste. The external aspects of the different cookies subjected to sensory evaluation are shown in Figure 3.

Parallel to the sensory test, another test was carried out to measure the degree of neophobia of the consumers who participated in the sensory test to assess whether the inclusion of novel ingredients such as chia or poppy could determine the preferences of consumers towards cookies that do not contain these ingredients.

The average value of the neophobia of the consumers who participated in the sensory analysis was 30.26 on a scale of 10 to 70 and the results are similar to the ones obtained by [38] that stated that the Spanish food neophobia mean value was 31.74.

The results obtained in the sensory evaluation for the external aspect, the texture and the taste are shown in Table 7. The changes in the color of the cookies elaborated with defatted flours of nuts and seeds did not seem to affect the consumers’ preferences, since the external appearance values of cookies elaborated with nut and seed flours and oils were similar or even higher (AW) than the values obtained by the control cookie. Color is an important parameter in judging baked cookies that not only reflects the ingredients used but also provides information about the formulation and the quality [39].

However, regarding the taste and the texture, the inclusion of chia (AWC) and poppy (AWP) flour in the formulation of the cookies were least preferred compared with the control sample, AW and AWB (*p* < 0.05). Similar results were obtained by [40] in a study carried out with burgers supplemented with chia and poppy seed oils and flours that obtained the lowest values on these parameters; this may be due to the presence of fiber and the characteristic flavor of that seeds. In addition, the lower scores in the reformulated cookies with a higher content of nut flours may be related to the higher moisture content they have presented and a lower crispy texture.

On the other hand, the control sample and the AWB sample obtained the higher results on taste and texture, differentiating from the other samples due to the presence of butter because it confers flavor and smell, contributing to the palatability, which is the reason why the use of butter is extended in the food industry in bakery products, pastries and toppings. Although the cookies elaborated from butter obtained the best results in texture and flavor, the other samples have also obtained high scores on both parameters, which indicates the good acceptance of all the batches by the consumers, (over 1.5) in a 9-point scale, where −4 implies extremely dislike, 0 neither like nor dislike and +4 extremely like.

Regarding the food neophobia results, it should be noted that non-neophobic consumers are the ones who give higher scores to the taste and texture of cookies elaborated using the traditional composition. Neophobic consumers’ aversions to trying new products makes them give even lower scores to the traditional ones, because when they are faced with a product they do not know, these consumers tend to systematically give lower values.

## 4. Conclusions

Cookies reformulated with almond, walnut, chia and poppy seed oils and flours are a nutritive alternative to traditional cookies that can be consumed also by celiac people as they do not contain any wheat in the formula. From the physical point of view, the reformulated cookies presented darker colors compared with the control sample, although it does not seem to be important for consumer evaluation. The control sample showed a lower expansion factor due to the presence of gluten in the formulation, and the texture of the cookies that contained flours and oils from seeds and nuts presented a softer texture compared with the control, which is linked to a lower acceptability when sensory tests are performed.

In nutritional terms, the resultant cookies had an increase in protein, fiber and fat content, with a reduction in the carbohydrate content, which represents an improvement in the nutritional characteristics of cookies. However, the energy value did not change significantly.

From the sensory point of view, the addition of poppy and chia flour resulted in lower values for taste and texture, but in general all the batches were well accepted by consumers since all evaluations were in the positive range. The lowest values of all the sensory characteristics evaluated corresponded to the neophobic consumers, who tend to reject foods with new ingredients that they are not used to.

The results obtained meet the demands of today’s society, as people are more concerned about the impact of food on health. Thus, it is important to develop new formulas containing functional components such as nuts and seeds capable of positively inducing the control and prevention of certain diseases, in addition to satisfying an increasing demand for gluten-free products. The inclusion of novel ingredients such as seed and nut oils and defatted flours, even in low concentrations, may lead to an increase in the nutrients needed by the human diet.

## Figures and Tables

**Table 1:** Nutritional composition of the flours.

	Chia	Poppy	Almond	Walnut
Moisture (%)	7.52 ± 0.15	8.01 ± 0.13	8.36 ± 0.59	11.39 ± 0.41
Ash (%)	6.17 ± 0.90	11.01 ± 0.14	6.10 ± 0.58	4.88 ± 0.34
Protein (% dw)	29.99 ± 1.82	28.05 ± 0.25	45.46 ± 6.77	37.89 ± 3.25
Total dietary fibre (% dw)	35.15 ± 1.45	41.02 ± 0.00	5.43 ± 0.50	3.76 ± 0.20
Fat (% dw)	11.67 ± 0.23	11.52 ± 0.18	21.11 ± 2.86	13.34 ± 4.12
Remaining carbohydrates (% dw)	17.02 ± 0.95	8.40 ± 0.41	21.90 ± 4.60	40.13 ± 6.19

**Table 2:** Chemical composition of the oils.

	Chia	Poppy	Almond	Walnut
C16:0 Palmitic acid	6.36 ± 0.03	8.29 ± 0.08	6.62 ± 0.05	5.97 ± 0.01
C16:1 Palmitoleic acid	0.04 ± 0.00	0.11 ± 0.01	0.50 ± 0.01	0.11 ± 0.00
C17:0 Margaric acid	0.04 ± 0.01	0.04 ± 0.00	0.10 ± 0.00	0.05 ± 0.00
C18:0 Stearic acid	2.83 ± 0.00	1.85 ± 0.08	2.06 ± 0.01	2.39 ± 0.01
C18:1n9 Oleic acid	4.98 ± 0.08	14.07 ± 0.08	70.82 ± 0.18	14.47 ± 0.02
C18:2n6 Linoleic acid	17.78 ± 0.12	74.80 ± 0.21	19.16 ± 0.08	61.51 ± 0.12
C18:3n3 α-Linolenic acid	67.66 ± 0.15	0.66 ± 0.04	0.10 ± 0.00	15.14 ± 0.02
C20:0 Arachidic acid	0.14 ± 0.01	0.07 ± 0.00	0.10 ± 0.00	0.08 ± 0.00
∑SFA	9.51 ± 0.04	10.31 ± 0.10	9.22 ± 0.09	8.49 ± 0.08
∑MUFA	5.05 ± 0.08	14.24 ± 0.09	71.32 ± 0.22	14.79 ± 0.09
∑PUFA	85.44 ± 0.10	75.45 ± 0.18	19.26 ± 0.12	76.65 ± 0.21

**Table 3:** Flour and fat source used in the formulation of the cookies elaborated. The percentage of flour and fat refers to the total quantity of flour and fat used.

Cookie ID	Flour		Fat	
	Ingredient	%	Ingredient	%
Control	Wheat	100	Butter	100
AWP	Maize	50	Walnut Poppy	70 30
	Almond	33		
	Walnut	14		
	Poppy	3		
AW	Maize	60	Walnut	100
	Almond	28		
	Walnut	12		
AWC	Maize	60	Walnut Almond	50 50
	Almond	23		
	Walnut	12		
	Chia	5		
AWB	Maize	82	Butter Walnut	50 50
	Walnut	12		
	Almond	6		

**Table 4:** Items of the food neophobia scale.

Item	Statements
1	I am constantly sampling new and different foods (R)
2	I do not trust new foods
3	I do not know what a food is, I will not try it
4	I like foods from different cultures (R)
5	Ethnic food looks weird to eat
6	At dinner parties, I will try new foods
7	I am afraid to eat things I have never had before (R)
8	I am very particular about the foods I eat
9	I will eat almost anything
10	I like to try ethnic restaurants (R)

(R) indicates negatively worded items for which scores were reversed in order to calculate the scale score.

**Table 5:** Dimensions of the samples.

	Diameter (mm)	Thickness (mm)	Spread factor
Control	45.769 ± 0.93 ^c^	8.932 ± 0.59 ^a^	7.70 ± 0.44 ^d^
AWP	49.130 ± 0.63 ^a^	7.192 ± 0.20 ^b^	10.25 ± 0.28 ^b^
AW	49.291 ± 0.68 ^a^	7.464 ± 0.43 ^b^	9.88 ± 0.47 ^c^
AWC	47.914 ± 1.07 ^b^	6.056 ± 0.91 ^c^	11.91 ± 1.40 ^a^
AWB	48.436 ± 0.92 ^b^	6.852 ± 0.51 ^c^	10.64 ± 0.66 ^b^

Control—wheat flour (100%) and butter (100%); AWP—maize flour (50%), almond flour (33%), walnut flour (14%), poppy flour (3%), walnut oil (70%) and poppy oil (30%); AW—maize flour (60%), almond flour (28%), walnut flour (12%) and walnut oil (100%); AWC—maize flour (60%), almond flour (23%), walnut flour (12%), chia flour (5%), almond oil (50%) and walnut oil (50%); AWB—maize flour (82%), walnut flour (12%), almond flour (6%), butter (50%) and walnut oil (50%). Different letters in the same column show significant differences (*p* < 0.05).

**Table 6:** Proximate composition of cookies elaborated with flours and oil from nuts and seeds.

	Control	AWP	AW	AWC	AWB
Moisture (%)	5.8 ± 0.2 ^c^	8.4 ± 0.4 ^b^	9.0 ± 0.2 ^b^	10.6 ± 0.5 ^a^	7.6 ± 0.3 ^bc^
Proteins (%)	9.31 ± 0.28 ^b^	15.44 ± 0.77 ^a^	14.94 ± 0.30 ^a^	14.63 ± 0.73 ^a^	9.88 ± 0.29 ^b^
Crude fiber (%)	0.38 ± 0.01 ^c^	1.58 ± 0.08 ^b^	1.30 ± 0.03 ^b^	2.33 ± 0.11 ^a^	0.89 ± 0.03 ^c^
Crude fat (%)	19.77 ± 0.59 ^b^	26.41 ± 1.32 ^a^	26.50 ± 0.53 ^a^	24.18 ± 1.21 ^a^	21.78 ± 0.65 ^b^
Total carbohydrates (%)	70.24 ± 2.11 ^a^	55.93 ± 2.80 ^b^	56.46 ± 1.13 ^b^	59.06 ± 2.95 ^b^	67.02 ± 2.01 ^a^
Digestive carbohydrates (%)	69.86 ± 2.10 ^a^	54.35 ± 2.72 ^b^	55.16 ± 1.10 ^b^	56.73 ± 2.83 ^b^	66.13 ± 1.98 ^a^
Energy value (Kcal/100 g dm)	496 ± 15	523 ± 26	524 ± 11	512 ± 26	504 ± 15

Control—wheat flour (100%) and butter (100%); AWP—maize flour (50%), almond flour (33%), walnut flour (14%), poppy flour (3%), walnut oil (70%) and poppy oil (30%); AW—maize flour (60%), almond flour (28%), walnut flour (12%) and walnut oil (100%); AWC—maize flour (60%), almond flour (23%), walnut flour (12%), chia flour (5%), almond oil (50%) and walnut oil (50%); AWB—maize flour (82%), walnut flour (12%), almond flour (6%), butter (50%) and walnut oil (50%). Different letters in the same line show significant differences (*p* < 0.05).

**Table 7:** Sensory analysis and consumer segmentation attending to the Food Neophobia Scale.

Sample	Total Consumers Valuation	Consumer Segmentation	
		Neophobics	Non-Neophobics
Aspect			
Control	2.21 ± 1.18 ^b^	1.72 ± 1.13 ^b^	2.54 ± 1.14 ^a^
AWP	2.16 ± 1.31 ^b^	1.83 ± 1.29	2.50 ± 1.22
AW	2.70 ± 0.86 ^a^	2.44 ± 0.78 ^b^	2.96 ± 0.81 ^a^
AWC	2.09 ± 1.19 ^b^	1.94 ± 1.21	2.25 ± 1.19
AWB	2.31 ± 1.19 ^b^	1.67 ± 1.24 ^b^	3.04 ± 0.91 ^a^
Texture			
Control	2.81 ± 1.18 ^a^	2.33 ± 1.46 ^b^	3.17 ± 0.82 ^a^
AWP	0.65 ± 0.78 ^c^	0.50 ± 0.79	0.79 ± 0.78
AW	1.21 ± 1.09 ^b^	1.33 ± 1.41	1.13 ± 0.80
AWC	1.19 ± 1.03 ^b^	0.94 ± 1.96	1.38 ± 1.10
AWB	2.40 ± 1.00 ^a^	2.33 ± 0.97	2.46 ± 1.06
Taste			
Control	2.93 ± 0.99 ^a^	2.56 ± 1.10 ^b^	3.21 ± 0.83 ^a^
AWP	1.12 ± 1.07 ^c^	0.67 ± 1.03 ^b^	1.50 ± 0.98 ^a^
AW	1.49 ± 1.18 ^c^	1.22 ± 1.00	1.71 ± 1.30
AWC	1.58 ± 1.20 ^c^	1.00 ± 1.03 ^b^	2.08 ± 1.10 ^a^
AWB	2.09 ± 1.21 ^b^	1.56 ± 1.34 ^b^	2.46 ± 0.98 ^a^

Control—wheat flour (100%) and butter (100%); AWP—maize flour (50%), almond flour (33%), walnut flour (14%), poppy flour (3%), walnut oil (70%) and poppy oil (30%); AW—maize flour (60%), almond flour (28%), walnut flour (12%) and walnut oil (100%); AWC—almond flour (23%), walnut flour (12%), chia flour (5%), almond oil (50%) and walnut oil (50%); AWB—maize flour (82%), walnut flour (12%), almond flour (6%), butter (50%) and walnut oil (50%). In the first column (total consumers) different letters show significant differences in the consumer evaluation of the same attribute for the five different samples (ANOVA, Duncan test, *p* < 0.05). On the second and third columns (consumer segmentation) different letters on the same row show significant differences for the same attribute in the same sample for the two consumer segments (*t*-test, *p* < 0.05).
